# Understanding Barriers to Routine HIV Screening: Knowledge, Attitudes, and Practices of Healthcare Providers in King County, Washington

**DOI:** 10.1371/journal.pone.0044417

**Published:** 2012-09-06

**Authors:** Alexandra Shirreffs, David P. Lee, Jsani Henry, Matthew R. Golden, Joanne D. Stekler

**Affiliations:** 1 Department of Health Services, University of Washington, Seattle, Washington, United States of America; 2 Department of Medicine, University of Washington, Seattle, Washington, United States of America; 3 Department of Epidemiology, University of Washington, Seattle, Washington, United States of America; 4 Public Health - Seattle & King County, Seattle, Washington, United States of America; The University of Hong Kong, China

## Abstract

**Objective:**

In 2006, the Centers for Disease Control and Prevention (CDC) recommended routine HIV screening in healthcare settings for persons between 13 and 64 years old. In 2010, the Washington Administrative Code (WAC) was changed to align testing rules with these recommendations. We designed this survey to ascertain the current state of HIV testing and barriers to routine screening in King County, Washington.

**Methods:**

Between March 23 and April 16, 2010, a convenience sample of healthcare providers completed an online survey. Providers answered true-false and multiple choice questions about national recommendations and the WAC, policies in their primary clinical settings, and their personal HIV testing practices. Providers were asked to agree or disagree whether commonly reported barriers limited their implementation of routine HIV screening.

**Results:**

Although 76% of the 221 respondents knew that the CDC recommended routine HIV screening for persons regardless of their risk, 99 (45%) providers reported that their primary clinical setting had a policy to target testing based on patient risk factors. Forty-four (20%) providers reported that their primary clinical setting had a policy of routine HIV screening, 54 (25%) reported no official policy, and 15 (7%) did not know whether a policy existed. Only 11 (5%) providers offer HIV testing to all patients at initial visits. When asked about barriers to routine screening, 57% of providers agreed that perception that their patient population is low risk limits the number of HIV tests they perform. Only 26 (13%) providers agreed that concern about reimbursement posed a barrier to testing.

**Conclusions:**

Most providers participating in this survey continue to target HIV testing, despite knowledge of national recommendations. Efforts are still needed to educate providers and policymakers, clarify the recent WAC revisions, and implement structural changes in order to increase HIV testing in Washington State.

## Introduction

In 2006, the Centers for Disease Control and Prevention (CDC) recommended that healthcare providers should shift away from targeting HIV testing based on risk factors and move towards a strategy of routine screening for all persons between the ages of 13 and 64 [1]. This was in response to findings that an estimated 21% of HIV-infected individuals in the United States are unaware of their HIV status [2], and many others are diagnosed late in infection [3], [4]. Routine screening has potential advantages over targeted testing because HIV-infected persons frequently access care prior to diagnosis [3], [5], [6] and routine screening may identify persons who are unaware of their risk or who do not disclose participation in risky behaviors if they are asked.

In 2009, Public Health - Seattle & King County (PHSKC) estimated that approximately 10–15% of the 7000–8000 persons living with HIV in King County, Washington were unaware of their HIV status [7]. In January 2010, the Washington Administrative Code (WAC 246–100–207) was revised to facilitate routine HIV screening by aligning state HIV testing rules with the 2006 CDC recommendations [**Table 1**]. Healthcare providers are no longer required to evaluate behavioral or clinical HIV risk factors prior to testing, provide pre-test risk-reduction counseling, or document consent for HIV testing. Under the new WAC, patients must be informed that HIV testing will be performed unless they opt out, and they must have an opportunity to ask questions. Post-test counseling is not required for persons with a non-reactive HIV test.

**Table 1:** 2006 CDC Recommendations and the Washington Administrative Code (WAC) rules for HIV testing.

	CDC Recommendations [1]	WAC Prior to 2010	2010 WAC Revisions
Consent	• General consent for medical careshould be considered sufficient toencompass consent for HIV testing.• Patients should be informed thattesting will be performed unless theydecline (opt-out screening).• If a patient declines an HIV test,this decision should be documentedin the medical record.	• Obtain or ensure explicit verbal or written consent, and document the consent.	• Obtain informed consent, separately or as part of consent for a battery of other routine tests.• Specifically inform patients that HIV testing is included, verbally or in writing.
Pre-test counseling	• Prevention counseling should not be required with HIV diagnostic testingor as part of HIV screening programs inhealth care settings.• Prevention counseling is strongly encouraged for persons at high riskfor HIV in settings in which risk behaviorsare assessed routinely (e.g. STD clinics)but should not have to belinked to HIV testing.	• Evaluate behavioral and clinical risk factorsfor HIV.• Explicitly provide information including:the benefits of testing, dangers of HIV, waysHIV is transmitted and prevented, andavailability of anonymous testing.• Recommend and offer or refer for pre-test counseling any person who requests it or is determined to be at increased risk.	• Offer patients the opportunity to ask questions and decline testing.
Post-test counseling		• Provide or refer for other prevention,support, or medical services.• Provide or ensure referral for post-test counseling if the test is positive foror suggestive of HIV infection.	• Provide or ensure post-test counseling or referral for persons with HIV tests that are positive or suggestive of HIV infection.

We designed this survey to ascertain the knowledge, attitudes, and practices of healthcare providers in King County regarding routine HIV screening, the CDC recommendations, and the 2010 WAC revisions. We were particularly interested in identifying any residual barriers that would prevent implementation of routine HIV screening.

## Methods

The survey was available online on SurveyGizmo between March 23 and April 16, 2010. We recruited a convenience sample of healthcare providers through key personnel at the largest primary care networks in King County (Group Health Cooperative, Healthpoint, Neighborcare Health, PHSKC clinics, the Polyclinic, and the University of Washington Medicine Neighborhood Clinics). Key personnel emailed the survey link to their provider networks along with a brief explanation of purpose. In order to oversample providers likely to be serving populations with higher HIV prevalence, we distributed the link on three listservs focused on local epidemiology, infectious diseases, and HIV and sexually transmitted infections (STIs). The survey was sent a single time.

Eligible individuals self-reported that they 1) were a physician, physician assistant, or nurse practitioner with authority to order HIV tests and 2) regularly provide direct patient care in King County to HIV-negative persons between the ages of 13 and 64. The University of Washington Human Subjects Division evaluated this activity and determined that it was not subject to review.

The anonymous, self-administered survey [**Appendix S1**] was adapted from publicly available instruments [8]–[10] and collected information on the demographics, medical specialty, and practice location of participants. Participants were specifically asked whether they subspecialize in infectious diseases, sexual transmitted diseases, HIV, or family planning services. Providers were asked one multiple choice question about the 2006 CDC recommendations and five true-false questions about the 2010 WAC revision. After each question, providers were given the option of viewing the correct response and explanation. Additional multiple choice questions asked providers about policies in their primary clinical locations. Providers were given a list of potential barriers to the implementation of routine HIV screening and asked whether they agreed, disagreed, or were undecided as to whether the statements reflected “factors that prevent [them] from offering routine HIV screening in [their] practice and/or limit the number of tests that [they] are able to do.”

We designed this survey in order to collect data about knowledge, attitudes, and practices regarding HIV testing; identify current barriers to routine HIV screening; and plan for future interventions. We present results primarily as descriptive analyses, with univariate regression analyses using STATA software (StataCorp LP, College Station, TX), where appropriate.

## Results

The survey was accessed 396 times, and 221 responses were analyzed. We excluded results from persons who were not physicians, physician’s assistants, or nurse practitioners (n = 40); did not provide direct patient care to HIV-negative persons between the ages of 13 and 64 (n = 110); did not continue beyond initial eligibility questions (n = 18); or reported that the zip code of their primary clinical practice was outside of King County (n = 7). Demographics of providers and characteristics of providers’ practices are shown in **Table 2**. The median age of providers was 53 (interquartile range, IQR, 43–59) years. A total of 73 (33%) providers reported practicing in a field relevant to HIV testing including 17 in HIV care, 38 in STIs, 24 in infectious diseases, and 48 who work in family planning services.

**Table 2:** Demographics of providers and characteristics of provider practices.

		N	%
Gender	Male	77	39
	Female	120	61
Race/ethnicity	Hispanic	5	3
	Caucasian	168	87
	Black/African-American	5	3
	Native American/Alaskan Native	1	1
	Asian	10	5
	Other	3	2
	Mixed	7	4
Degree	Doctor of Medicine	145	74
	Nurse Practitioner	32	16
	Physician’s Assistant	9	5
	Doctor of Naturopathic Medicine	7	4
	Doctor of Osteopathic Medicine	2	1
Specialty	Family Medicine	83	42
	Internal Medicine	45	23
	Pediatrics	27	14
	Obstetrics and Gynecology	18	9
	Emergency Medicine	9	5
	Surgery	1	1
	Other	15	8
Work Setting	Ambulatory Clinic	149	76
	Hospital - Inpatient	43	22
	Hospital - Emergency Department	11	6
	Hospital - Outpatient	23	12
	Other	6	3

When asked to select the one response that best summarized the 2006 CDC recommendations, 62 (30%) providers responded that the CDC recommended testing for all persons between 13 and 64 years of age regardless of risk factors, 11 (5%) agreed only with the statement that HIV testing should be performed if the population prevalence of HIV is greater than 0.1%, 36 (17%) responded that the CDC recommended testing patients who report HIV risk factors, three (1%) thought the CDC recommended testing patients who display signs or symptoms of AIDS, and 97 (46%) providers responded correctly that the CDC recommendations included all of the previous statements. We considered persons to be aware of recommendations for routine HIV screening if they selected either “all of the above” or the first answer only, as this statement reflected the change in the 2006 recommendations. There was a trend towards greater awareness of the recommendations among providers who had a subspeciality related to HIV compared to those who did not (OR 2.0, 95% CI 1.0–4.1, p = .07), but there were no associations between awareness of these recommendations and provider gender, age, or race/ethnicity.

When asked about the 2010 WAC revisions, 174 (86%) providers answered correctly that specific informed consent was still required for HIV testing, 131 (65%) providers answered correctly that written consent was not required by the WAC, and 125 (61%) providers answered correctly that the WAC requires documentation only when a pregnant woman refuses consent for HIV testing. Only 40 (20%) providers responded correctly that the WAC does not require provision of post-test counseling for all clients. There were no significant statistical associations between provider characteristics and WAC knowledge. Almost half (47%) of providers thought that the recent WAC changes would increase the number of patients they tested for HIV or the number of HIV tests they would perform.

When asked about HIV testing policies, 99 (45%) providers reported that their primary clinical practice has a policy recommending targeted testing based on a patient’s risk factors, and 44 (20%) reported that their practice has a policy of routine HIV screening. Providers with a subspecialty related to HIV were more likely to report that their practice had a policy of routine HIV screening (29%, OR 2.2, 95% CI 1.1–4.3, p = .02) compared to those who practiced in other fields. The remaining providers reported no policy (n = 54, 25%) or did not know whether a policy existed in their practice (n = 15, 7%).

Most providers (n = 183, 91%) use blood-based antibody tests for HIV screening, 19 (9%) use rapid HIV antibody tests, three (1%) screen for acute HIV infection with pooled nucleic acid amplification testing (NAAT), and 21 (10%) use HIV NAAT to test persons with symptoms consistent with the acute retroviral syndrome. **Table 3** shows the number of HIV tests ordered by providers in the previous six months and the number of new diagnoses made in the previous year.

**Table 3:** HIV tests ordered and new diagnoses of HIV infection, by provider type.

HIV Tests Ordered in Last 6 Months	All Providers N = 219	HIV-related specialty^1^ N = 73
None	26 (12%)	3 (4%)
1–5	62 (28%)	20 (27%)
6–10	47 (22%)	16 (22%)
11–24	39 (18%)	15 (21%)
25 or more	45 (21%)	19 (26%)
**New HIV Diagnoses in Last Year**		
Never	28 (13%)	7 (10%)
None in last 12 months	151 (69%)	46 (63%)
1 individual	21 (10%)	8 (11%)
2–5 individuals	17 (8%)	10 (14%)
6 or more individuals	2 (1%)	2 (3%)

1 Includes providers specializing in HIV, sexually transmitted infections, infectious diseases, and/or family planning.

**Table 4** shows the proportions of surveyed providers who agreed that proposed barriers reflected factors that prevent them from offering routine HIV screening or limit the number of tests that they perform. The most commonly cited barrier to routine HIV screening - endorsed by 119 (57%) providers - was the perception that their patients are at low risk for HIV infection. This response was associated with female gender of providers (OR 1.9, 95% CI 1.1–3.5, p = .03) and was less common among providers who worked in the zip codes where the Public Health STD Clinic and two Ryan White funded HIV clinics are located (OR 0.4, 95% CI 0.2–0.7, p = .002); this barrier was not otherwise associated with provider age, race/ethnicity, specialty, or practice setting. Few providers (3%) responded that they did not feel comfortable discussing HIV, sexual behavior, or drug use with patients. Also notably, only 26 (13%) respondents reported concern about reimbursement as a barrier to HIV testing. Finally, providers who did not know that written consent was not required by the WAC were more likely to agree that the consent process was too time consuming and/or burdensome (OR 2.8, 95% CI 1.4–5.6, p = .003).

**Table 4:** Response to putative barriers to routine HIV screening.

	All subjects N = 221	HIV-related specialty N = 73
	n (%)	n (%)
I think the risk of HIV among my patient population is low.	119 (57%)	37 (51%)
Nothing, I conduct routine HIV testing for all adolescent and adult patients.	69 (34%)	32 (45%)
The pre-test or risk reduction counseling is too time consuming and/or burdensome.	63 (31%)	12 (17%)
The consent process for HIV testing is too time consuming and/or burdensome.	46 (22%)	7 (10%)
I do not understand the legal procedures or implications associated with HIV testing.	42 (20%)	7 (10%)
I am concerned about language barriers.	39 (19%)	12 (17%)
I do not have resources to assure an HIV-positive diagnosis will occur smoothly withappropriate follow-up.	37 (18%)	13 (18%)
I do not have enough time to conduct HIV tests.	35 (17%)	5 (7%)
I am concerned I cannot provide enough information for questions the patientmight have about HIV testing.	34 (16%)	9 (12%)
I do not have enough experience providing pre-test or risk reduction counseling.	29 (14%)	5 (7%)
I am concerned about reimbursement.	26 (13%)	12 (17%)
I do not think my patients would feel comfortable discussing HIV, sex behaviors,or drug use with me.	21 (10%)	3 (4%)
I do not have a private space to do testing.	7 (3%)	0
I do not feel comfortable discussing HIV, sex behaviors, or drug use with my patients.	6 (3%)	1 (1%)
HIV testing is prohibited in my practice.	1 (0%)	0

Numbers and proportions indicate providers who strongly or somewhat agreed that the statement reflected a factor that prevents them from offering routine HIV screening in their practice and/or limits the number of HIV tests that they are able to do.

1 Includes providers specializing in HIV, sexually transmitted infections, infectious diseases, and/or family planning.

## Discussion

Although most of the providers who participated in this survey knew of the CDC recommendations, only 20% practice in a setting with a policy of routine HIV screening, and few providers offer HIV testing to all of their patients. These findings are consistent with results from similar surveys [9], [10] and with data showing that the proportion of Americans who have ever tested for HIV increased only slightly from 40% in 2006 to 45% in 2009 [11]. Similarly, responses here regarding educational, logistical, and policy barriers to implementation of routine HIV screening were consistent with what has been outlined elsewhere [8]. In our survey, the most commonly endorsed barrier was the perception of providers that their patients are low risk. Although this perception may be valid to some degree, routine HIV screening is thought to be cost-effective when the population prevalence is greater than 0.1% [12], [13]. In Seattle, the HIV prevalence identified through pilot studies of routine HIV screening in a community clinic and in the emergency department and inpatient medicine service of the publicly-funded county hospital ranged from 0.4 to 0.9% [Drs. Joanne Stekler and Ann Kurth, unpublished data]. Not surprisingly, providers who practice in the zip codes where these pilot studies were done were less likely to endorse perception of risk as a barrier to screening. Our findings suggest that healthcare providers need further education regarding the rationale for local implementation of the recommendations, including the cost-effectiveness of routine HIV screening and prevalence of HIV infection in King County.

Since 2006, Washington and 24 other states (including Washington, D.C.) have made legislative changes to reduce legal barriers to routine HIV screening [14], [15]. Changes to the WAC in 2010 eliminated the need to perform a risk assessment, provide pre-test risk-reduction counseling, and document consent except for refusal by a pregnant woman. Our findings indicate that providers would also benefit from education regarding the current regulations around HIV testing and the new emphasis on opt-out testing, which has been credited with increasing the uptake of prenatal screening from 25–83% to 71–98% [16]. Despite the WAC changes, some local healthcare institutions continue to maintain stricter counseling, consent, and documentation requirements due to risk management concerns. Efforts to educate providers will be ineffective unless institutional policies can also be aligned with the CDC recommendations.

There also continues to be debate about the CDC recommendations themselves. Some question the shift to routine screening when emergency department screening programs identify only a modest increase in number of cases using routine screening compared to appropriately-targeted testing, and at greater cost [17], [18]. In 2006, the United States Preventative Services Task Force (USPSTF) recommended neither for or against routine HIV screening, a grade C recommendation [19]. Although most providers we surveyed were not concerned about levels of reimbursement for HIV testing, this issue is highly relevant to the success of routine HIV screening, as the Affordable Care Act will require coverage of preventive services that have received a USPSTF A or B rating. The USPSTF is anticipated to re-issue recommendations around routine HIV screening within the next year.

Structural interventions will also be needed in order to ensure that any testing program is sustainable. Programs that have dedicated staff to provide rapid antibody testing are likely to increase the uptake of HIV testing [20], but the lower sensitivity and the personnel costs of such strategies may not be advantageous in settings with competing priorities. Hybrid models could be potential solutions [21]. Strategies that automate testing through electronic reminders and computer-generated reports could also have impact with minimal ongoing effort [22]. Here we should look once again to prenatal HIV screening programs for guidance. Implementation of prenatal HIV screening was likely successful because HIV tests could be added to a panel of laboratory tests that were already routine and acceptable. Although unlikely to have universal coverage, we should consider whether it is possible to package HIV tests with other screening tests for adults.

Our sampling strategies and lack of study funding are the greatest limitations of this evaluation. We were most interested in surveying primary care providers, obstetricians/gynecologists, providers of emergency and urgent care, and other persons likely to have contact to undiagnosed persons with HIV infection. Unfortunately, the Washington State Department of Health did not collect email addresses of licensed providers until recently, and snail mail addresses could not be restricted by specialty. We elected to perform the study as described but are unable to estimate the number of persons reached out of the approximately 23,000 licensed providers in Washington or the response rate, although we suspect that it was low. We were also not able to provide an incentive for participants. It is therefore unclear whether our results are representative of healthcare providers in King County. If providers with a particular interest in this issue were more likely to participate, it is possible that we overestimated knowledge of the CDC recommendations and potentially the level of routine HIV screening in King County.

In summary, HIV testing remains one of the most effective HIV prevention interventions because persons who are unaware of their infection may contribute to over half of all new infections [23], individuals newly diagnosed with HIV infection will alter their behaviors to reduce the risk of HIV transmission to others [24]–[26], and receipt of antiretroviral therapy can both improve individual health and decrease onward transmission [27]. For these reasons, the National HIV/AIDS Strategy has focused public health efforts on the HIV care cascade, from diagnosis to linkage to care and antiretroviral treatment. Targeted, risk-based testing has been insufficient to date, and efforts are needed to promote routine HIV screening, increase the frequency of testing among high-risk persons, and reduce the time interval in which HIV-infected persons are unaware of their HIV status. The 2006 CDC recommendations and subsequent legislative changes were but the first steps. Successful implementation of routine HIV screening in Washington State and nationally will likely require additional provider education, policy changes in local institutions, and structural interventions to create sustainable programs for the future.

## Supporting Information

Appendix S1 Self-administered Survey.
(PDF)

## References

[1] Branson BM, Handsfield HH, Lampe MA, Janssen RS, Taylor AW, et al (2006) Revised recommendations for HIV testing of adults, adolescents, and pregnant women in health-care settings. MMWR Recomm Rep, 55: 1–17; quiz CE11–14. 16988643

[2] HIV prevalence estimates–United States, 2006 (2008) MMWR Morb Mortal Wkly Rep. 57: 1073–1076. 18830210

[3] Late HIV testing - 34 states, 1996–2005 (2009) MMWR Morb Mortal Wkly Rep. 58: 661–665. 19553901

[4] Ogbuanu IU, Torres ME, Kettinger L, Albrecht H, Duffus WA (2009) Epidemiological characterization of individuals with newly reported HIV infection: South Carolina, 2004–2005. Am J Public Health 99 Suppl 1S111–117. 18048784 10.2105/AJPH.2006.104323 PMC2724932

[5] Liddicoat RV, Horton NJ, Urban R, Maier E, Christiansen D, et al (2004) Assessing missed opportunities for HIV testing in medical settings. J Gen Intern Med 19: 349–356. 15061744 10.1111/j.1525-1497.2004.21251.x PMC1492189

[6] Jenkins TC, Gardner EM, Thrun MW, Cohn DL, Burman WJ (2006) Risk-based human immunodeficiency virus (HIV) testing fails to detect the majority of HIV-infected persons in medical care Settings. Sex Transm Dis 33: 329–333. 16547450 10.1097/01.olq.0000194617.91454.3f

[7] HIV/AIDS Epidemiology Unit, Public Health – Seattle & King County and the Infectious Disease and Reproductive Health Assessment Unit, Washington State Department of Health. HIV/AIDS Epidemiology Report, 1st Half 2009: Volume 74. Available: http://www.kingcounty.gov/healthservices/health/communicable/hiv/epi.aspx. Accessed 2011 March 1.

[8] Burke RC, Sepkowitz KA, Bernstein KT, Karpati AM, Myers JE, et al (2007) Why don’t physicians test for HIV? A review of the US literature. Aids 21: 1617–1624. 17630557 10.1097/QAD.0b013e32823f91ff

[9] Jain CL, Wyatt CM, Burke R, Sepkowitz K, Begier EM (2009) Knowledge of the Centers for Disease Control and Prevention’s 2006 routine HIV testing recommendations among New York City internal medicine residents. AIDS Patient Care STDS 23: 167–176. 19866534 10.1089/apc.2008.0130

[10] Korthuis PT, Berkenblit GV, Sullivan LE, Cofrancesco J Jr, Cook RL, et al (2011) General internists’ beliefs, behaviors, and perceived barriers to routine HIV screening in primary care. AIDS Educ Prev 23: 70–83. 21689038 10.1521/aeap.2011.23.3_supp.70 PMC3196638

[11] Vital signs: HIV testing and diagnosis among adults–United States, 2001–2009 (2010) MMWR Morb Mortal Wkly Rep. 59: 1550–1555. 21124295

[12] Sanders GD, Bayoumi AM, Sundaram V, Bilir SP, Neukermans CP, et al (2005) Cost-effectiveness of screening for HIV in the era of highly active antiretroviral therapy. N Engl J Med 352: 570–585. 15703422 10.1056/NEJMsa042657

[13] Paltiel AD, Weinstein MC, Kimmel AD, Seage GR 3rd, Losina E, *et al* (2005) Expanded screening for HIV in the United States–an analysis of cost-effectiveness. N Engl J Med 352: 586–595. 15703423 10.1056/NEJMsa042088

[14] Wolf LE, Donoghoe A, Lane T (2007) Implementing routine HIV testing: the role of state law. Plos One 2: e1005. 17925853 10.1371/journal.pone.0001005 PMC1994587

[15] Neff S, Goldschmidt R (2011) Centers for Disease Control and Prevention 2006 human immunodeficiency virus testing recommendations and state testing laws. JAMA 305: 1767–1768. 21540419 10.1001/jama.2011.564

[16] HIV testing among pregnant women–United States and Canada, 1998–2001 (2002) MMWR Morb Mortal Wkly Rep. 51: 1013–1016. 12458916

[17] Haukoos JS, Hopkins E, Conroy AA, Silverman M, Byyny RL, et al (2010) Routine opt-out rapid HIV screening and detection of HIV infection in emergency department patients. JAMA 304: 284–292. 20639562 10.1001/jama.2010.953

[18] d’Almeida KW, Kierzek G, de Truchis P, Le Vu S, Pateron D, et al (2012) Modest public health impact of nontargeted human immunodeficiency virus screening in 29 emergency departments. Arch Intern Med 172: 12–20. 22025095 10.1001/archinternmed.2011.535

[19] Screening for Human Immunodeficiency Virus: focused update of a 2005 systematic evidence review for the U.S. Preventive Services Task Force. AHRQ Pub No. 07–0597–1-EF (April 2007). In. 20722143

[20] Anaya HD, Hoang T, Golden JF, Goetz MB, Gifford A, et al (2008) Improving HIV screening and receipt of results by nurse-initiated streamlined counseling and rapid testing. J Gen Intern Med 23: 800–807. 18421508 10.1007/s11606-008-0617-x PMC2517869

[21] Hutchinson AB, Farnham PG, Lyss SB, White DA, Sansom SL, et al (2011) Emergency department HIV screening with rapid tests: a cost comparison of alternative models. AIDS Educ Prev 23: 58–69. 21689037 10.1521/aeap.2011.23.3_supp.58

[22] Goetz MB, Hoang T, Henry SR, Knapp H, Anaya HD, et al (2009) Evaluation of the sustainability of an intervention to increase HIV testing. J Gen Intern Med 24: 1275–1280. 19798538 10.1007/s11606-009-1120-8 PMC2787938

[23] Marks G, Crepaz N, Janssen RS (2006) Estimating sexual transmission of HIV from persons aware and unaware that they are infected with the virus in the USA. Aids 20: 1447–1450. 16791020 10.1097/01.aids.0000233579.79714.8d

[24] Colfax GN, Buchbinder SP, Cornelisse PG, Vittinghoff E, Mayer K, et al (2002) Sexual risk behaviors and implications for secondary HIV transmission during and after HIV seroconversion. Aids 16: 1529–1535. 12131191 10.1097/00002030-200207260-00010

[25] The Voluntary HIV-1 Counseling and Testing Efficacy Study Group (2000) Efficacy of voluntary HIV-1 counselling and testing in individuals and couples in Kenya, Tanzania, and Trinidad: a randomised trial. Lancet 356: 103–112. 10963246

[26] Marks G, Crepaz N, Senterfitt JW, Janssen RS (2005) Meta-analysis of high-risk sexual behavior in persons aware and unaware they are infected with HIV in the United States: implications for HIV prevention programs. J Acquir Immune Defic Syndr 39: 446–453. 16010168 10.1097/01.qai.0000151079.33935.79

[27] Cohen MS, Chen YQ, McCauley M, Gamble T, Hosseinipour MC, et al (2011) Prevention of HIV-1 infection with early antiretroviral therapy. N Engl J Med 365: 493–505. 21767103 10.1056/NEJMoa1105243 PMC3200068

