# Supplementary material for: Understanding Barriers to Routine HIV Screening: Knowledge, Attitudes, and Practices of Healthcare Providers in King County, Washington
Source: PLoS One. 2012 Sep 6;7(9):e44417. doi: 10.1371/journal.pone.0044417 (PMC3435280; doi:10.1371/journal.pone.0044417)
Supplement: Appendix S1 — Self-administered Survey. (PDF) [file pone.0044417.s001.pdf]

PHSKC HIV Testing Survey: Knowledge, Attitudes and Practices

Page One

This anonymous survey is intended to collect information about HIV testing attitudes and practices. Results will be used by Public Health - Seattle & King County's HIV/AIDS Program to:

Determine current attitudes and practices regarding HIV testing in King County, WA;

Determine awareness of January 2010's changes to testing guidelines outlined in the Washington Administrative Code; and

Identify barriers that prevent providers from implementing routine HIV screening

This survey contains 25 questions in 3 sections.

Estimated Time: 10 - 15 MINUTES

Questions about this survey can be directed to the survey administrator,
Alex Shirreffs at alexshir@u.washington.edu or 206.205.6105.

Eligibility Q1

SECTION 1: ELIGIBILITY QUESTIONS

The following series of questions will determine your eligibility to participate in this survey.

1. Do you regularly provide direct patient care to HIV-negative individuals between the ages of 13 and 64 years old? (Required)

- ◯ Yes
- ◯ No

Created using SurveyGizmo

Eligibility Q2

2. Are you a doctor, physician's assistant, or nurse practitioner with authority to order HIV tests at your practice? (Required)

- ○ Yes
- ○ No

Created using SurveyGizmo

Section 2

SECTION 2: PRACTICE & KNOWLEDGE QUESTIONS

This series of questions will help Public Health – Seattle & King County understand the HIV testing practices of local providers and assess their knowledge of HIV testing guidelines recommended by the U.S. Centers for Disease Control and Prevention (CDC) and the HIV testing rules outlined in the Washington Administrative Code (WAC).

Testing Practices

3. Is there an HIV testing policy at your primary work location?

- ○ Yes, routine testing*
- ○ Yes, targeted testing based on a patient’s risk factors
- ○ No, my practice does not have a HIV testing policy
- ○ HIV testing is prohibited in my practice
- ○ I don't know
- ○ Other ____________

*According to the CDC,"'Routine counseling and testing' is defined as a policy to provide these services to all clients after informing them that testing will be done. Except where testing is required by law, individuals have the right to decline to be tested without being denied health care or other services."

4. Which of these describe how you personally approach HIV testing with your patients (check all that apply)?

- ☐ I test all of my adolescent and adult patients at their first visit
- ☐ I test patients who report HIV risk factors
- ☐ I test patients who have signs or symptoms of HIV/AIDS.
- ☐ I test any patient who asks for an HIV test
- ☐ I never order HIV tests
- ☐ Other ____________

How Many Tests/How Many Positive Results

5. In the past SIX MONTHS, approximately how many times did you personally order an HIV test?

- ○ None
- ○ 1 to 5
- ○ 6 to 10
- ○ 11 to 24
- ○ 25 or more

6. In the past TWELVE MONTHS approximately how many patients did you diagnose as HIV-positive?

- ○ None in the past year
- ○ 1
- ○ 2 to 5 individuals
- ○ 6 or more individuals
- ○ I have never in my career newly diagnosed a patient with HIV infection

Barriers to Testing

7. There are many reasons why providers may not offer routine HIV screening in their practices.

Please rate how strongly you agree or disagree with the statements below in regard to factors that prevent you from offering routine HIV screening in your practice and/or limit the number of tests that you are able to do:

	Strongly disagree	Disagree	Agree	Strongly agree	Undecided
Nothing, I conduct routine HIV testing for all adolescent and adult patients.	◯	◯	◯	◯	◯
I do not have enough time to conduct HIV tests.	◯	◯	◯	◯	◯
I think that the consent process for HIV testing is too time consuming and/or burdensome.	◯	◯	◯	◯	◯
I think that pre-test or risk reduction counseling is too time consuming and/or burdensome.	◯	◯	◯	◯	◯
I do not have enough experience providing pre-test or risk reduction counseling.	◯	◯	◯	◯	◯
I do not understand the legal procedures or implications associated with HIV testing (e.g.: reporting HIV-positive cases or counseling requirements).	◯	◯	◯	◯	◯
I do not have resources to assure an HIV-positive diagnosis will occur smoothly with appropriate follow-up.	◯	◯	◯	◯	◯
I am concerned about reimbursement.	◯	◯	◯	◯	◯
I am concerned I cannot provide enough information for questions the patient might have about HIV testing.	◯	◯	◯	◯	◯
I am concerned about language barriers.	◯	◯	◯	◯	◯
I do not feel comfortable discussing HIV, sex behaviors, or drug use with my patients.	◯	◯	◯	◯	◯
I do not think my patients would feel comfortable discussing HIV, sex behaviors, or drug use with me.	◯	◯	◯	◯	◯
I do not have a private space to do testing.	◯	◯	◯	◯	◯
I think the risk of HIV among my patient population is low.	◯	◯	◯	◯	◯
HIV testing is prohibited in my practice.	◯	◯	◯	◯	◯

Please specify any other reasons why you do not conduct routine HIV testing in your practice.

Created using SurveyGizmo

CDC Knowledge

In September 2006, CDC released its Revised Recommendations for HIV Testing of Adults, Adolescents, and Pregnant Women in Health-Care Settings. These recommendations were released to increase the proportion of HIV-infected persons who are aware of their HIV status.

The following question will help us understand how familiar you are with the CDC recommendations.

8. Check one answer that best reflects the CDC's recommendations:

- ◯ Test all patients between 13-64 years of age for HIV, regardless of risk factor history
- ◯ Test all patients for HIV if the prevalence of HIV in your community is greater than .1%
- ◯ Test those patients who report HIV risk factors
- ◯ Test those patients who display signs or symptoms of AIDS
- ◯ All of the above

Would you like to see the correct answer with additional information about the CDC testing guidelines?

- ◯ Yes, take me to the answer!
- ◯ No thanks, I'd like to continue with the survey.

Created using SurveyGizmo

Answer Key: CDC

Because 1 out of 5 people infected with HIV are not aware of their status, the CDC revised its guidelines to promote increased HIV screening.   All of the above is an appropriate response to Question 3, because all of the answers are core elements of the 2006 guidelines for screening in health care settings:

Screening for HIV Infection

In all health-care settings, screening for HIV infection should be performed routinely for all patients aged 13--64 years. Health-care providers should initiate screening unless prevalence of undiagnosed HIV infection in their patients has been documented to be <0.1%. In the absence of existing data for HIV prevalence, health-care providers should initiate voluntary HIV screening until they establish that the diagnostic yield is <1 per 1,000 patients screened, at which point such screening is no longer warranted.

Repeat Screening

Health-care providers should subsequently test all persons likely to be at high risk for HIV at least annually. Persons likely to be at high risk include injection-drug users and their sex partners, persons who exchange sex for money or drugs, sex partners of HIV-infected persons, and MSM or heterosexual persons who themselves or whose sex partners have had more than one sex partner since their most recent HIV test.

Diagnostic Testing for HIV Infection

All patients with signs or symptoms consistent with HIV infection or an opportunistic illness characteristic of AIDS should be tested for HIV.

Clinicians should maintain a high level of suspicion for acute HIV infection in all patients who have a compatible clinical syndrome and who report recent high-risk behavior. When acute retroviral syndrome is a possibility, a plasma RNA test should be used in conjunction with an HIV antibody test to diagnose acute HIV infection.

The entire

Normal.dotm 0 0 1 15 88 University Of Washington 1 1 108 12.0 0 false 18 pt 18 pt 0 0 false false false

Revised recommendations for HIV testing of adults, adolescents, and pregnant women in health-care settings can be accessed here:

http://www.cdc.gov/mmwr/preview/mmwrhtml/rr5514a1.htm

WAC Knowledge

9. The following questions will help us understand how familiar you are with the Washington Administrative Codes (WAC) rules for HIV testing, counseling, and partner services. These rules were recently changed in mid-January 2010.

Please answer the following True/False questions about the current WAC rules:

	True	False	I Don't Know
The WAC requires informed consent for HIV testing.	○	○	○
The WAC requires written consent for HIV testing.	○	○	○
The WAC requires providers to offer post-test prevention counseling to all patients.	○	○	○
The WAC requires providers to document a pregnant patient's refusal of an HIV test.	○	○	○
The WAC requires opt-out testing.*	○	○	○

* The CDC defines opt-out screening as “performing HIV screening after notifying the patient that 1) the test will be performed and 2) the patient may elect to decline or defer testing. Assent is inferred unless the patient declines testing.”

Would you like to see the correct answers with additional information about the WAC HIV testing rules?

- ○ Yes, take me to the answer!
- ○ No thanks, I'd like to continue with the survey.

WAC Answers

The Washington Administrative Code's new HIV testing, counseling, and partner services rules offer flexibility to health care providers and local health jurisdictions. The rules remove specific requirements for HIV testing, counseling, and partner services no longer recommended by CDC. The rules provide health care providers with the flexibility to offer HIV testing to all individuals.

According to the revised WAC rules of January 2010:

Informed consent is required? True. Informed consent can be obtained separately or with general consent for care. Patients must be specifically informed verbally or in writing that a test for HIV is included.

Written consent is required? False. Although the WAC do not require written consent, requirements of your institution(s) may vary.

All patients must receive post-test counseling? False. Post-test counseling is only required for patients whose HIV test is positive or indeterminate.

If a pregnant patient refuses an HIV test it must be documented? True. For pregnant women only, documentation is required only when a woman refuses an HIV test.

Opt-out testing is required? False. The new rules do not require, but certainly encourage, providers to implement opt-out testing.

Official rule language can be accessed here:

http://apps.leg.wa.gov/documents/laws/wsr/2010/01/10-01-082.htm

Created using SurveyGizmo

Impact of WAC

10. The Washington Administrative Code (WAC) changed in January 2010 to align more closely with the CDC's 2006 HIV Testing Guidelines. Providers will not be required to conduct detailed pre-test counseling but they will still be required to inform patients when an HIV test is being conducted, giving patients the option to decline or "opt-out."

	Very Unlikely	Somewhat Unlikely	Somewhat Likely	Very Likely	Undecided	Not Applicable
Will these changes increase your HIV testing practices?	○	○	○	○	○	○

Please share any concerns you have about the WAC changes.

Created using SurveyGizmo

Risk Factor Screening Questions

11.

Which of the following information do you routinely collect to assess a patient’s risk of infection with the HIV or Hepatitis C viruses?

	Yes	No
Country of birth	○	○
History of injection drug use	○	○
History of methamphetamine or stimulants use	○	○
For men, whether they have ever had sex with another man	○	○
For women, pregnancy history	○	○
For men and women, number of recent sexual partners and use of condoms	○	○
STD history	○	○
Hepatitis B status	○	○
Hepatitis C status	○	○
History of blood transfusion or organ transplant prior to 1985	○	○

Which HIV Test

12. Which HIV tests do you use for HIV screening (check all that apply)?

- ☐ Standard HIV antibody tests on blood
- ☐ Rapid HIV antibody tests: finger prick or oral fluids
- ☐ Pooled HIV RNA testing
- ☐ HIV RNA testing for symptomatic persons
- ☐ I don't know
- ☐ We do not offer HIV testing at my practice

13. Have you attended any meeting, lectures, or other information sessions regarding HIV testing within the last 12 months?

- ◯ Yes
- ◯ No

Created using SurveyGizmo

Hep C Questions

The CDC estimates that a quarter of people with HIV are co-infected with Hepatitis C. Because HCV is a bloodborne virus, injection drug users (IDUs) are at particularly high risk of co-infection - 50%-90% of IDUs are estimated to be infected with both HIV and HCV. Though it is less common, there are cases in which HCV is transmitted through sexual intercourse.

Because HIV and HCV have similar risk factors, the next two questions ask about your HCV testing practices.

14. Do you routinely test patients for Hepatitis C?

- ○ Yes
- ○ No

15. Which of these describe how you personally approach Hepatitis C (HCV) testing with your patients (check all that apply)?

- ☐ I test all of my adolescent and adult patients at their first visit
- ☐ I test patients who report risk factors including injection drug use or men who have sex with men
- ☐ I test patients who have signs or symptoms of HCV, including elevated liver function tests or signs of cirrhosis
- ☐ I test any patient who asks for an HCV test
- ☐ I never order HCV tests
- ☐ Other

Demographic: Zip/Provider Type

SECTION 3: DEMOGRAPHIC QUESTIONS

16. What is the ZIP code of the primary practice location where you provide direct patient care?

17. I am a:
- ◯ Nurse Practitioner
- ◯ Physician's Assistant
- ◯ M.D. or D.O.

Created using SurveyGizmo

Demographic: Practice Setting

18. Check one box that best describes your primary clinical specialty:

- ◯ Emergency Medicine
- ◯ Family Medicine
- ◯ Geriatrics
- ◯ Internal Medicine
- ◯ Obstetrics/Gynecology
- ◯ Pediatrics
- ◯ Surgery
- ◯ Other (specify) ________

19. Check the boxes that apply to the main sub-specialties you practice:

- ☐ Infectious diseases
- ☐ Sexually transmitted diseases, not including HIV
- ☐ HIV
- ☐ Other (specify) ________

20. Check the box(es) that best describes the work setting(s) where you provide direct patient care:

- ☐ Ambulatory Clinic or Office
- ☐ Hospital – Inpatient
- ☐ Hospital – Emergency Room
- ☐ Hospital – Outpatient
- ☐ Other (specify) ________

Demographic: Gender/Age/Race/Ethnicity

21. What is your gender?
- ○ Male
- ○ Female
- ○ Transgender

22. What is your age?

23. Are you of Spanish/Hispanic/Latino origin?
- ○ Yes
- ○ No

24. What do you consider your race to be:
- ☐ White
- ☐ Black/African-American
- ☐ Native American/Alaskan Native
- ☐ Asian
- ☐ Pacific Islander/Hawaiian Native
- ☐ Other

Final Comments

25. Please make any other comments you would like:
